# Parkin truncating variants result in a loss-of-function phenotype

**DOI:** 10.1038/s41598-019-52534-6

**Published:** 2019-11-06

**Authors:** Mariana Santos, Sara Morais, Conceição Pereira, Jorge Sequeiros, Isabel Alonso

**Affiliations:** 1 UnIGENe, IBMC - Institute for Molecular and Cell Biology, i3S - Instituto de Investigação e Inovação em Saúde, Universidade do Porto, Porto, Portugal; 2 CGPP, IBMC - Institute for Molecular and Cell Biology, i3S - Instituto de Investigação e Inovação em Saúde, Universidade do Porto, Porto, Portugal; 3 ICBAS - Instituto de Ciências Biomédicas Abel Salazar, Universidade do Porto, Porto, Portugal

**Keywords:** Mitochondrial proteins, Mechanisms of disease, Parkinson's disease

## Abstract

Parkinson disease (PD) is the second most common neurodegenerative disorder. Most cases of PD are sporadic, while 5–10% have a known genetic basis. Variants in the *PARK2* gene are the most frequent cause of autosomal recessive juvenile-onset PD. *PARK2* encodes parkin, a multi-domain protein that functions as an ubiquitin E3 ligase. Numerous variants spanning all parkin domains have been identified, although the pathogenic relevance for several of those remains unclear. In this study, we aimed to functionally characterize two truncating parkin variants: N52Mfs*29, which is highly prevalent in the Portuguese and Spanish populations, and L358Rfs*77, recently identified in the Portuguese population. Our results indicate that both variants are prematurely degraded by the proteasome, even though proteins levels are still moderate. We also showed that they are aggregation-prone and lead to mislocalized parkin. Interestingly, the L358Rfs*77 variant is mislocalized to the nucleus, which was never reported for parkin variants. While N52Mfs*29 impaired self-ubiquitination activity, the L358Rfs*77 variant seemed to retain it. Both variants, however, fail to ubiquitinate p62 substrate and did not relocalize to depolarized mitochondria. Therefore, we conclude that parkin truncating variants cause loss of parkin function, thus showing their causative role in PD pathogenesis.

## Introduction

Parkinson Disease (PD) is the second most common neurodegenerative disorder, after Alzheimer’s Disease^1^. PD is clinically characterized by motor symptoms, including resting tremor, muscle rigidity, bradykinesia and postural instability. PD symptoms result mainly from a progressive loss of dopaminergic neurons in the *substantia nigra pars compacta*, associated with the accumulation of intracellular proteinaceous inclusions (Lewy bodies)^2^.

Most cases of PD are sporadic, with unknown aetiology, while 5–10% have a known underlying genetic basis. Moreover, several genes have been associated with inherited forms of Parkinsonism^3–5^. Variants in *PARK2* are the most frequent cause of autosomal recessive juvenile-onset parkinsonism (ARJP) worldwide. Exonic deletions in the *PARK2* gene, encoding parkin, were first reported in Japanese families with ARJP^6^. Since then, numerous variants have been identified throughout the sequence of this particularly large gene (1.35 Mb), including large rearrangements, small deletions/insertions and missense/nonsense variants^3,4,7^.

Parkin protein is a well-established RBR (RING-between-RING) type of ubiquitin E3 ligase with multiple domains: an N-terminal ubiquitin like domain (UBL), followed by two RING finger domains (RING0 and RING1), an in-between-RING finger domain (IBR), a linker domain termed repressor element of parkin (REP) and a C-terminal RING finger domain (RING2)^8–10^. Resolution of the crystal structure showed that parkin exists in an autoinhibitory state, thereby needing additional conformational changes, mediated by PINK1 phosphorylation, to be active^8–12^. Parkin ubiquitinates a wide variety of cytosolic and mitochondrial proteins, being capable of catalyzing different types of ubiquitination (K63, K48, K11 and K6 linkages). Furthermore, parkin also ubiquitinates itself, promoting its own degradation^13–17^. Thereby, parkin has been characterized as a multifunctional protein involved in many cellular processes, including control of mitochondrial integrity and mitophagy, regulation of apoptosis, transcription and synaptic function^18^.

Given the complex activation process of parkin and depending on the specific residue affected, disease-associated variants can affect parkin E3 ligase activity through different mechanisms. These variants can directly impair parkin activity or abolish translation of a functional protein, cause reduced solubility and enhanced aggregation, disturb protein folding and stability, and/or affect parkin ability to bind to cofactors and substrates^19–21^. Here we report the functional characterization of two parkin truncating variants: N52Mfs*29 that has a high prevalence in the Portuguese and Spanish populations^7,22^, although without a clear biochemical characterization; and L358Rfs*77 that was recently identified in the Portuguese population but has not been functionally characterized yet. Our study showed that each variant lead to misfolding and mislocalized parkin, being defective in the ubiquitination process and resulting in an apparent loss of parkin function.

## Results

### The N52Mfs*29 and L358Rfs*77 variants are degraded by the proteasome

**Figure 1:**
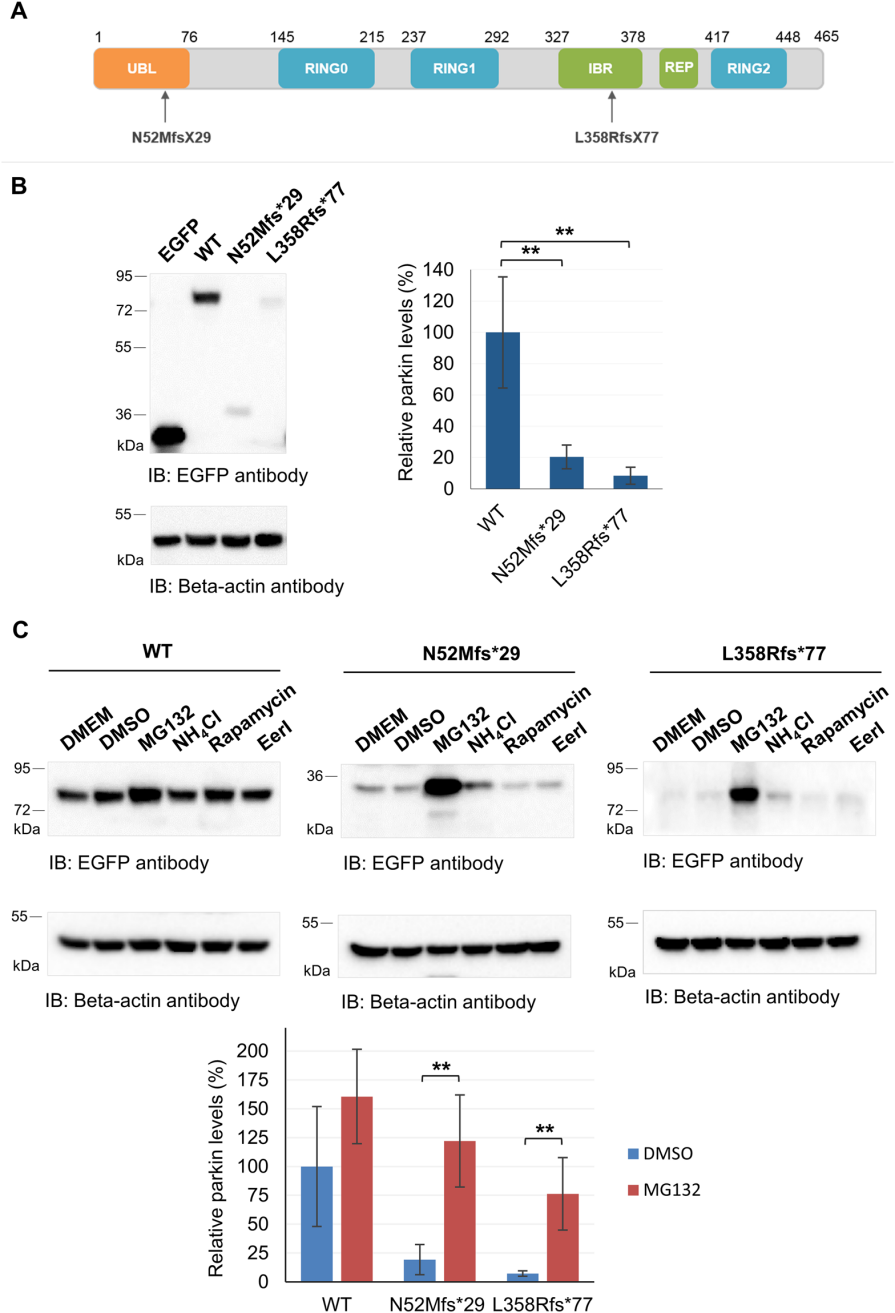
Parkin truncating variants have reduced protein expression. (**A**) Schematic representation of parkin (NP_004553) functional domains and location of the frameshift variants identified in Portuguese patients. (**B**) Analysis of protein expression of EGFP-tagged parkin clones in HEK293T cells by immunoblotting with anti-EGFP antibody. Beta-actin was used as loading control. Original blots are presented in Supplementary Fig. S2. Quantification data (graph) are presented as the mean ± SD of three independent experiments; **p < 0.01, compared with WT parkin (one-way ANOVA/Tukey); variance homogeneity assumption not fulfilled. (**C**) Identification of parkin proteolytic pathways. Cells were incubated with different drugs for 18 hours and cells lysates subjected to immunoblotting with anti-EGFP antibody. Beta-actin was used as loading control. Membranes were cut and incubated separately with either anti-EGFP or anti-Beta-actin antibodies. Quantification data (graph) of significant conditions (MG132 treatment) are presented as the mean ± SD of three independent experiments; **p < 0.01, compared with DMSO condition (one-way ANOVA/Tukey).

The p.N52Mfs*29 (c.155delA) parkin variant causes alteration of the open reading frame, which results in a premature stop codon, leading to the loss of most of the protein. This variant contains mostly the UBL domain of parkin^7,22^. The p.L358RfsX77 parkin variant (c.1072–1073delCTinsA), located in the IBR domain (Fig. 1A), is predicted to alter the open reading frame and consequently introduce a premature stop codon, leading to a truncated protein lacking the REP linker and the RING2 domain and part of the IBR domain^7^.

In order to understand the impact of these variants on the expression of parkin, we transfected HEK293T cells with plasmids containing N-terminal EGFP-tagged WT, N52Mfs*29 or L358Rfs*77 parkin variants, or control empty plasmid, followed by immunoblotting with anti-EGFP antibody or anti-beta-actin antibody (used as loading control). At 24 hours post-transfection, N52Mfs*29 and L358Rfs*77 showed significantly reduced protein levels when compared with WT parkin (Fig. 1B, about 80% and 92% decrease; p = 0.009 and 0.004, respectively). To note that, both N52Mfs*29 and L358Rfs*77 proteins presented the expected molecular weight of about 36 kDa (9 kDa from parkin plus 27 kDa from EGFP) and 77 kDa (50 kDa from parkin plus 27 kDa from EGFP). Quantitative real-time PCR showed no significant reduction on either EGFP-tagged N52Mfs*29 or L358Rfs*77 mRNA (Supplementary Fig. 1). Thereby, decreased levels of parkin variants are not the result of reduction or instability of the mRNA, but may result instead from decreased protein stability.

Therefore, we treated cells with different drugs to identify the proteolytic pathway responsible for the diminished levels of parkin protein variants. HEK293T cells were transfected with EGFP-tagged parkin (WT, N52Mfs*29 or L358Rfs*77), or control plasmid. 24 hours post-transfection, cells were treated for the following 18 hours with either MG132 (5 µM), rapamycin (200 nM), EerI (10 µM) or control vehicle (DMSO), or NH_4_Cl (15 mM) or control vehicle (DMEM). Treatment of cells with the proteasome inhibitor MG132 resulted in an increase in protein levels of WT parkin (Fig. 1C), but this was not significant (p = 0.117). Nonetheless, we observed a significant increase in protein levels of both N52Mfs*29 and L358Rfs*77, promoted by MG132 (p = 0.003 and p = 0.006 respectively). Inhibition of the proteasome with MG132 restored N52Mfs*29 levels, while L358Rfs*77 levels did not completely returned to WT parkin levels, possibly indicating that other proteolytic pathway may also be involved in its degradation. Nevertheless, inhibition of autophagy or ERAD, promoted by NH_4_Cl or EerI, respectively, and induction of autophagy by rapamycin did not significantly altered the levels of WT or parkin variants. These results show that N52Mfs*29 and L358Rfs*77 are preferentially targeted to the proteasome for degradation.

### The N52Mfs*29 and L358Rfs*77 variants alter the solubility and localization of parkin

**Figure 2:**
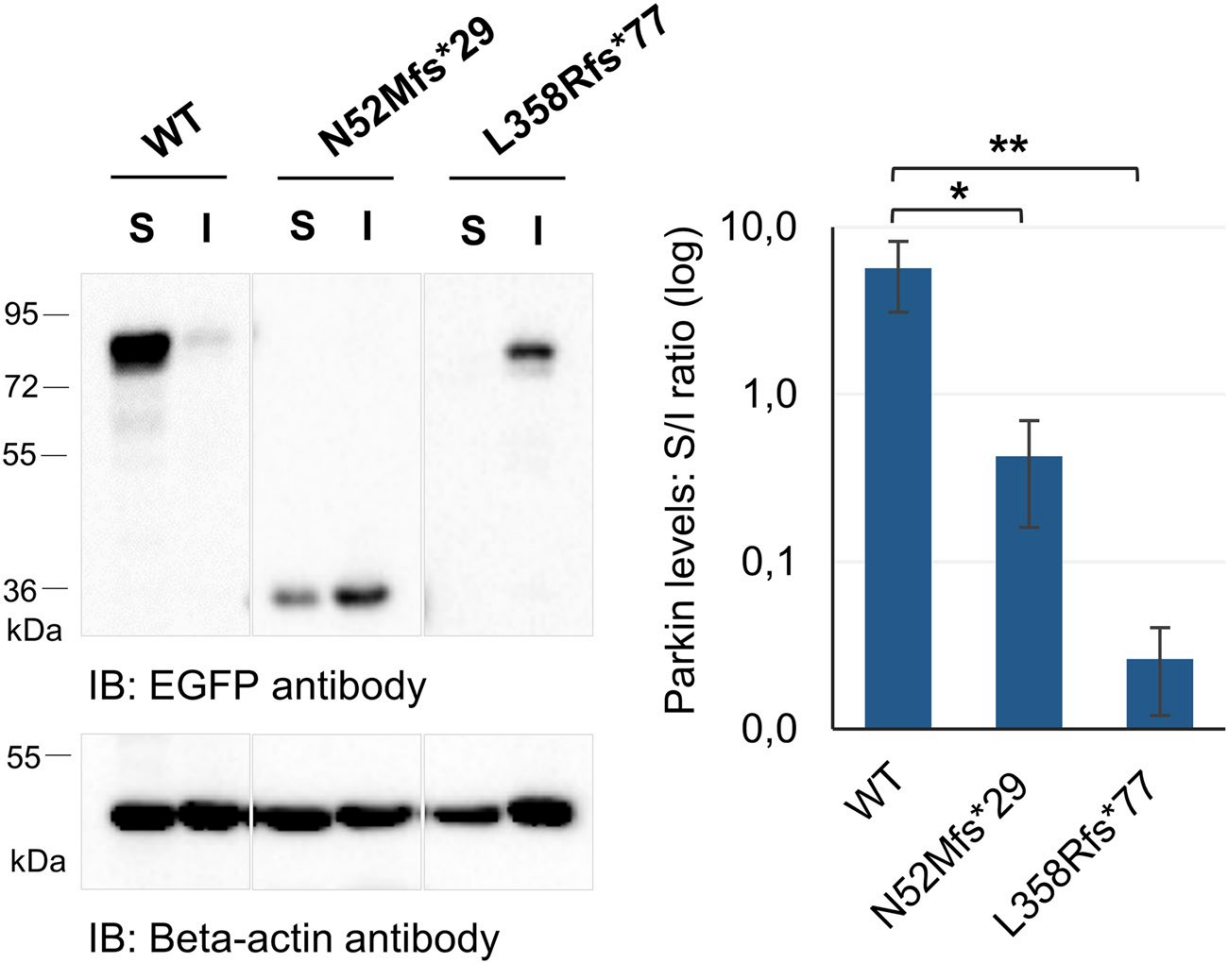
Parkin truncating variants have altered solubility properties. Equal amounts of soluble (S) and insoluble (I) fractions were loaded on gel followed by immunoblotting with anti-EGFP antibody. EGFP immunoblotting figures have different exposure times (original blots are presented in Supplementary Fig. S3). Quantification data (graph) are presented as the mean ± SD of three independent experiments; *p < 0.05, **p < 0.01, compared with WT parkin (one-way ANOVA/Tukey).

Previous studies reported that different missense/nonsense parkin variants show differential extractability by detergents due to altered localization and/or aggregate formation^14,21,23^. To address this issue, N52Mfs*29 or L358Rfs*77 variants expressed in HEK293T cells, as previously described, were compared with WT parkin for their ability to be extracted by 0.1% Triton X-100 detergent. Equal amounts of soluble and insoluble fractions were loaded on gel followed by immunoblotting with anti-EGFP antibody. The detergent solubility assay showed that, in comparison with WT parkin, there was a significant increase in the detergent-insoluble fraction when N52Mfs*29 or L358Rfs*77 variants were analyzed (Fig. 2; p = 0.012 and 0.008). Moreover, the L358Rfs*77 variant mostly adopted an insoluble conformation.

**Figure 3:**
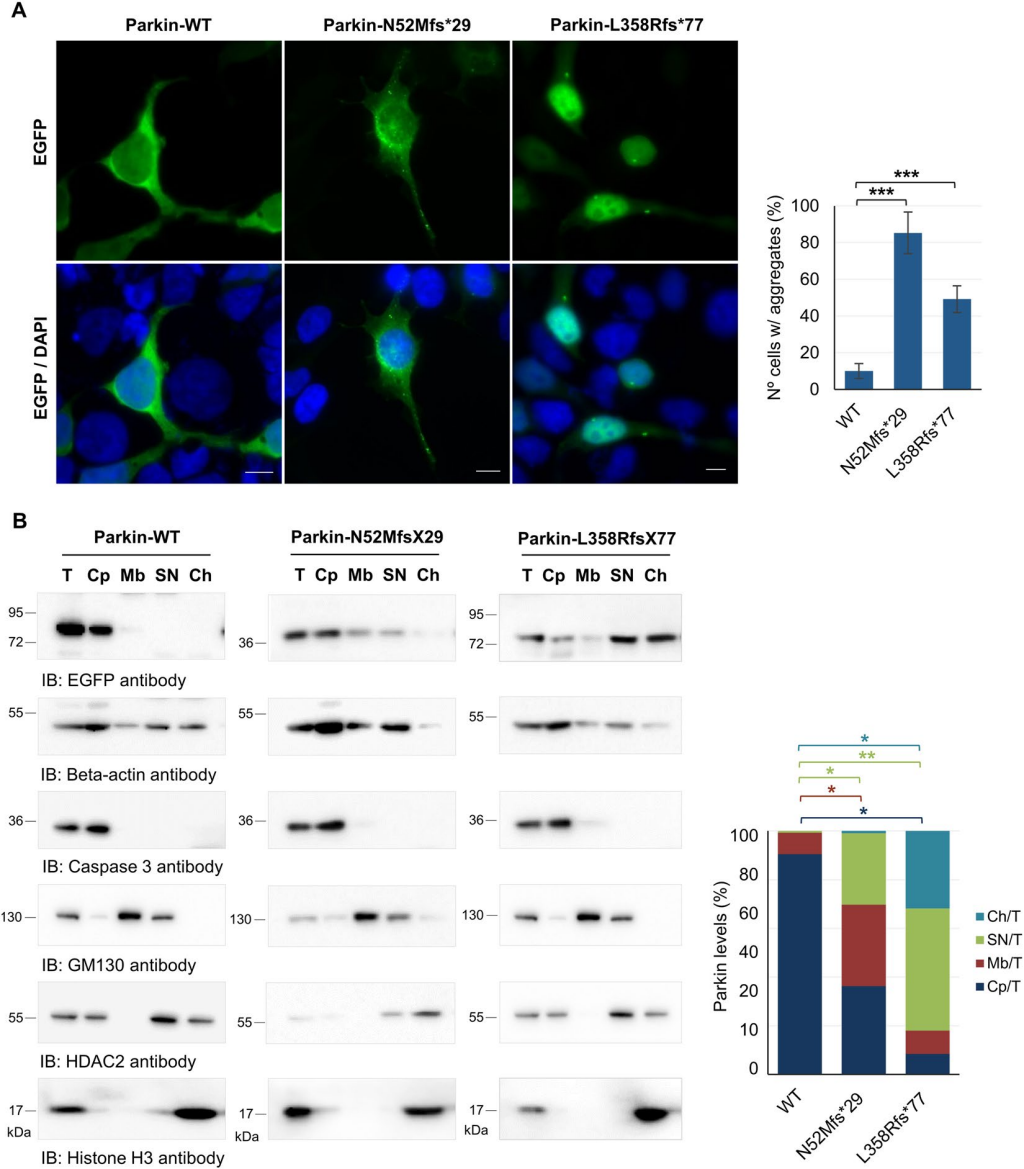
Truncating variants change the subcellular localization of parkin. (**A**) Localization of EGFP-tagged parkin clones in HEK293T cells. EGFP was directly visualized. Photographs were acquired using a Zeiss Axio Imager Z1 microscope. Bars, 10 μm. The percentage of cells with aggregates is also shown (graph). Data are presented as the mean ± SD of three independent experiments; ***p < 0.001 (ANOVA/Tukey). (**B**) Subcellular fractionation of HEK293T cells expressing parkin clones. Presence of parkin in each fraction was analyzed by immunoblotting with anti-EGFP antibody and normalized to the specific marker of each fraction. T, total fraction; Cp, cytoplasm fraction; Mb, membrane fraction; SN, soluble nuclear fraction; Ch, chromatin-bound nuclear fraction. Original blots are presented in Supplementary Fig. S4. Quantification data (graph) are presented as the mean ± SD of three independent experiments; *p < 0.05, **p < 0.01 (one-way ANOVA/Tukey).

The differential solubility of parkin variants lead us to study the subcellular localization of these proteins by fluorescence microscopy and subcellular fractionation. EGFP-tagged parkin clones were transfected in HEK293T cells for 24 hours and further prepared for fluorescence microscopy. EGFP-tagged proteins were directly visualized and DAPI was used to label the nucleus. WT parkin was homogenously located in the cytoplasm (Fig. 3A) with occasional parkin-positive aggregates, which were observed in about 10% of the total population of transfected cells. WT parkin seemed to be present also within the nucleus (Fig. 3A). In contrast, about 93% of cells transfected with N52Mfs*29 had parkin-positive aggregates in the cytoplasm (Fig. 3A). Moreover, N52Mfs*29 also appears to locate within the nucleus (Fig. 3A). Surprisingly, L358Rfs*77 seemed to be mainly located in the nucleus (Fig. 3A) and, only in a minor extent, in the cytoplasm. About 50% of cells transfected with L358Rfs*77 had parkin-positive aggregates in both the nucleus and cytoplasm.

The subcellular localization of WT parkin and variants was confirmed in HEK293T cells by subcellular protein fractionation. The fractionation procedure efficiency was controlled by immunoblotting using specific antibodies for each fraction isolated: beta-actin (total fraction), caspase 3 (cytoplasm fraction), GM130 (membrane fraction), HDCA2 (soluble nuclear fraction) and histone H3 (chromatin-bound nuclear fraction). Presence of parkin in each fraction was analyzed by immunoblotting with anti-EGFP antibody and normalized to the specific marker of the respective fraction. The enrichment of parkin in each fraction was determined by calculating the ratio between the amount of recovered parkin in each fraction and the amount of recovered parkin in whole cell lysates. WT parkin was mainly collected in the cytoplasm fraction (90.5%), while a smaller amount was detected in the membrane fraction (8.7%) (Fig. 3B). WT parkin was also present in the soluble nuclear fraction in a reduced amount (0.7%), but was barely detected in the chromatin-bound nuclear fraction (Fig. 3B). The N52Mfs*29 variant was collected in similar amount in the cytoplasm, membrane and nuclear fractions (36.2, 33.4, and 29.4%, respectively), while a smaller amount was detected in the chromatin-bound nuclear fraction (0.45%). When compared with WT parkin, the N52Mfs*29 variant caused a significant increase on the membrane and soluble nuclear fractions (Fig. 3B, graph; p = 0.049 and 0.032). Surprisingly, the L358Rfs*77 variant was predominantly collected in the soluble and chromatin-bound nuclear fractions (50.1 and 31.8%, respectively). This variant was also detected in the cytoplasm and membrane fractions in a reduced amount (8.4 and 9.5%, respectively) (Fig. 3B). When compared with WT parkin, the L358Rfs*77 variant caused a significant reduction in parkin levels in the cytoplasm and a significant increase on the soluble and chromatin-bound nuclear fractions (Fig. 3B, graph; p = 0.012, 0.004 and 0.041).

### The N52Mfs*29 and L358Rfs*77 variants impair parkin activity

**Figure 4:**
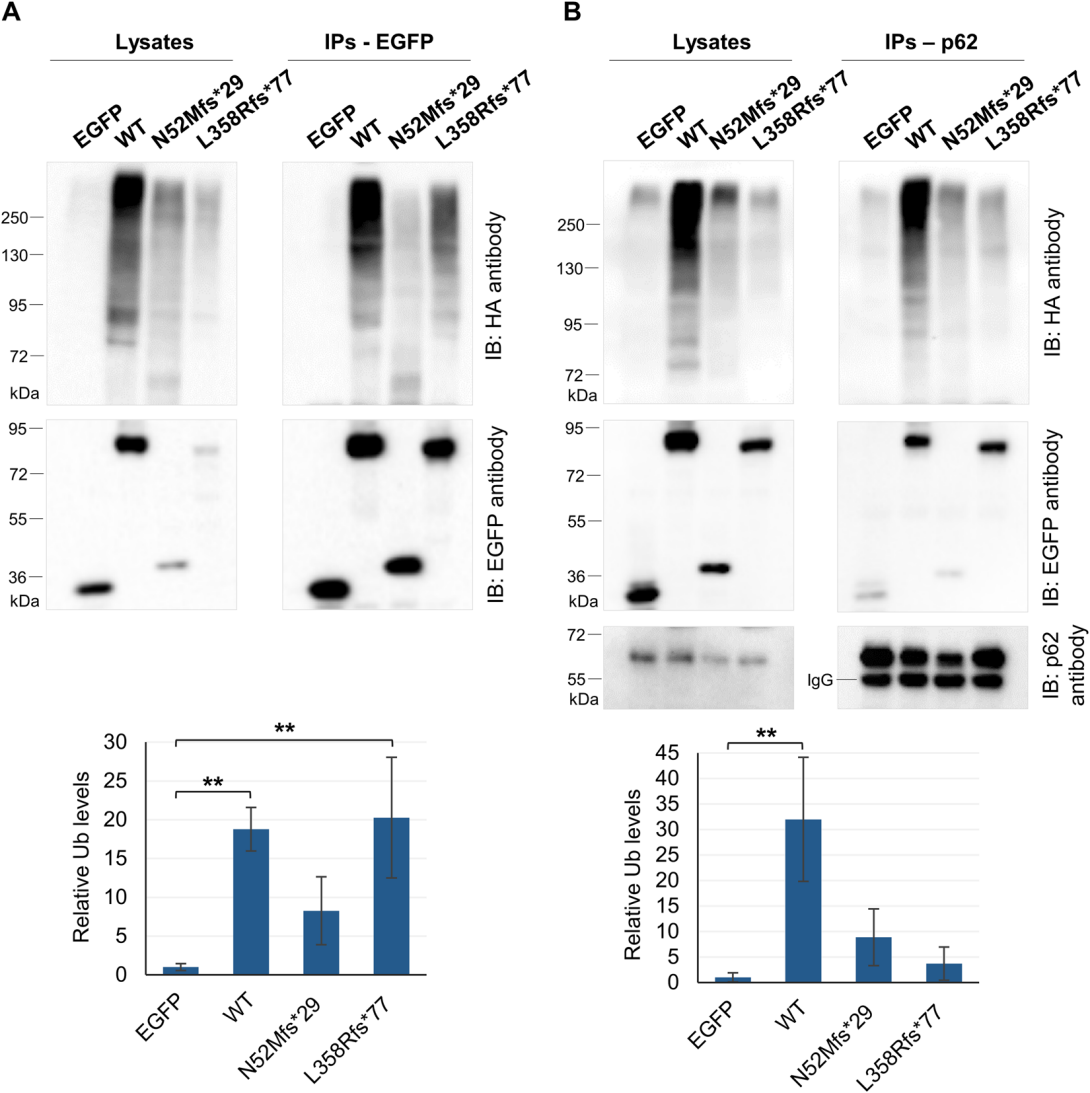
Parkin variants have different ubiquitination activity. (**A**) Co-immunoprecipitation of EGFP-tagged parkin clones with HA-tagged ubiquitin in HEK293T cells. Cell lysates were immunoprecipitated with anti-GFP antibody. Immunoprecipitates were subjected to immunoblotting with anti-HA antibody that revealed a characteristic ubiquitin smear corresponding to the amount of ubiquitination of each parkin clone. The blot was stripped and reprobed with anti-EGFP antibody. The same methodology was applied to whole cell lysates. (**B**) Co-immunoprecipitation of EGFP-tagged parkin clones with HA-tagged ubiquitin and endogenous p62 in HEK293T cells. Cell lysates were immunoprecipitated with anti-p62 antibody. Immunoprecipitates were immunoblotted with anti-HA antibody. Blot was stripped and reprobed with anti-EGFP and p62 antibodies. The same methodology was applied to whole cell lysates. p62 immunoblotting figures have different exposure times (original blots are presented in Supplementary Fig. S5). Quantification data (graphs, **A** and **B**) are presented as the mean ± SD of three independent experiments; **p < 0.01, compared with EGFP control plasmid (one-way ANOVA/Tukey); variance homogeneity assumption not fulfilled (graph A).

Parkin can promote its self-ubiquitination and this activity has been used to measure the enzymatic function of parkin^13,14,21,24^. Therefore, we have investigated whether the N52Mfs*29 and L358Rfs*77 variants impair parkin self-ubiquitination activity in cells. For that, we co-transfected EGFP-tagged parkin (WT, N52Mfs*29 or L358Rfs*77), or control plasmid, with HA-tagged ubiquitin in HEK293T cells. Subsequently, cell lysates were co-immunoprecipitated using a GFP-tag antibody and the immunoprecipitates analyzed for ubiquitin-positive smear by immunoblotting with an HA-tag antibody. Given the low expression of parkin variants (Fig. 1), compared with parkin WT and control plasmid, the ubiquitin (HA) immunoreactivity was quantified through the HA/EGFP immunoreactivity ratio. The immunoprecipitated WT parkin and the L358Rfs*77 variant showed a significant increase in HA immunoreactivity, compared with control plasmid, and a characteristic ubiquitin smear (Fig. 4A; p = 0.007 and 0.004, respectively), consistent with parkin self-ubiquitination activity. On the other hand, the immunoprecipitated N52Mfs*29 variant did not present a significant increase in HA immunoreactivity compared with control plasmid (Fig. 4A; p = 0.32), suggesting that this variant lacks self-ubiquitination activity.

Next, we examined whether the N52Mfs*29 and L358Rfs*77 variants affected the ability of parkin to ubiquitinate substrates in cells in the presence of the proteasome inhibitor MG132. We performed co-immunoprecipitations using p62 antibody followed by immunoblotting with HA-tag and EGFP-tag antibodies. As expected, HA immunoreactivity of immunoprecipitated p62 significantly increased in the presence of WT parkin, confirming that p62 is ubiquitinated by parkin (Fig. 4B; p = 0.003). On the other hand, HA immunoreactivity of immunoprecipitated p62 did not show a significant increase in the presence of neither N52Mfs*29 or L358Rfs*77 variants compared with control plasmid (Fig. 4B; p = 0.535 and 0.962, respectively), thus showing that these variants impair parkin enzymatic activity. Whole cell lysates were also loaded and membranes further immunoblotted (Fig. 4A,B) to show the levels of transfected EGFP-clones in each sample.

### Parkin redistribution to depolarized mitochondria

**Figure 5:**
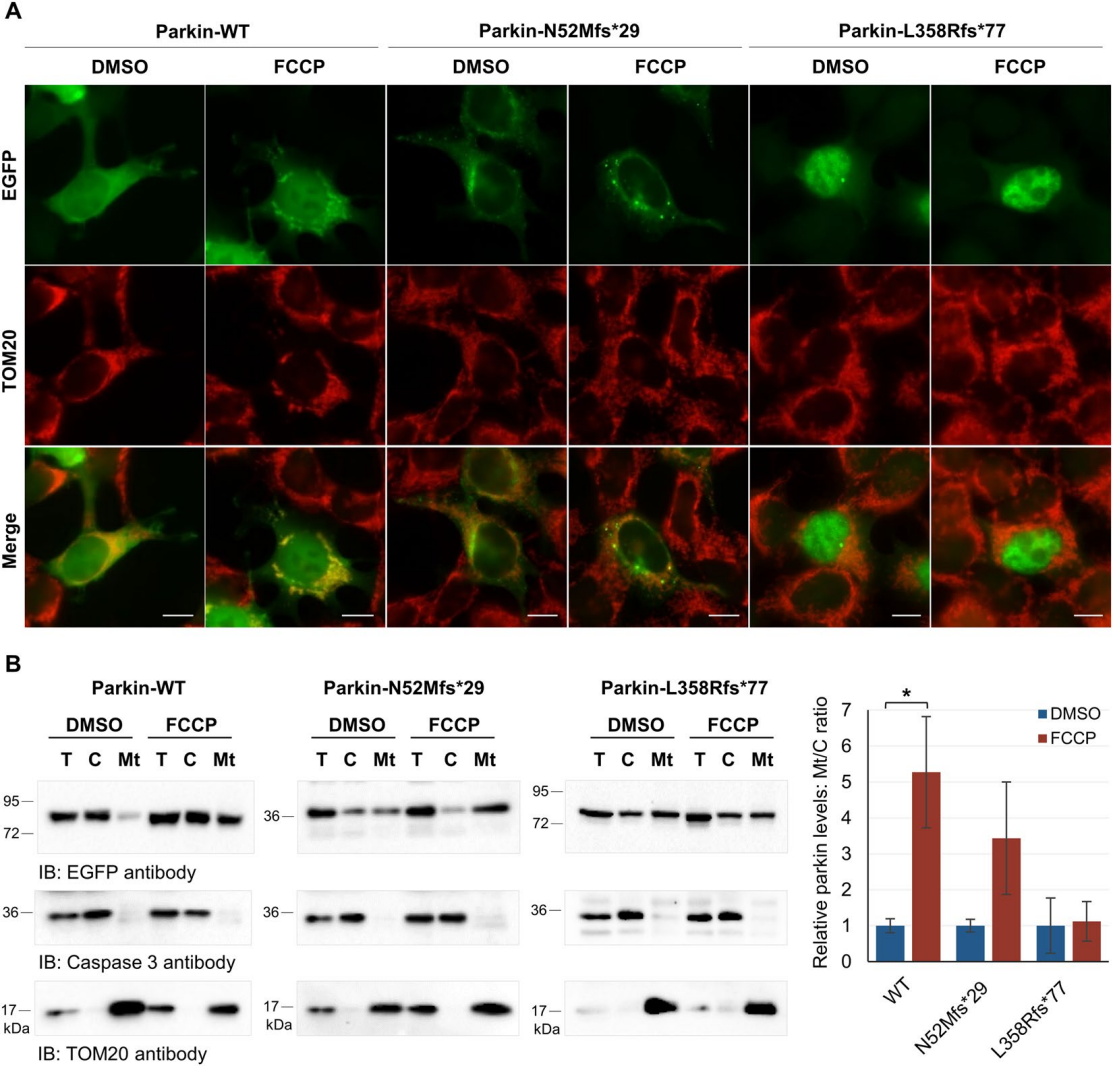
Only WT parkin is able to redistribute to depolarized mitochondria. (**A**) Immunofluorescence localization of EGFP-tagged parkin clones in HEK293T cells treated with FCCP or control vehicle DMSO. EGFP was directly visualized. TOM20 labelled mitochondria. Photographs were acquired using a Zeiss Axio Imager Z1 microscope. Bars, 10 μm. (**B**) Mitochondria isolation from HEK293T cells expressing parkin clones. Presence of parkin in cytosol (**C**) or mitochondria (Mt) fractions was analyzed by immunoblotting with anti-EGFP antibody and normalized to caspase 3 or TOM20, respectively. Original blots are presented in Supplementary Fig. S6. Quantification data (graph) are presented as the mean ± SD of three independent experiments; *p < 0.05, compared with control DMSO (two-tailed Student’s t-test).

Mitochondria dysfunction is an important contributor to PD pathogenesis. Particularly, parkin and PINK1 mediate autophagy of damaged mitochondria (mitophagy), a process that is impaired by PD-associated variants^25,26^. Translocation of parkin to mitochondria is essential in this process, thus we went on to test if parkin variants impair mitochondrial localization after mitochondria membrane depolarization using FCCP. HEK293T cells transfected with EGFP-tagged parkin clones were treated for 1 hour with either FCCP (30 µM) or control vehicle DMSO, and further prepared for fluorescence microscopy or mitochondria isolation experiments. WT parkin was diffusely distributed throughout the cytosol and occasionally seemed to overlap with mitochondria (labelled with TOM20) (Fig. 5A). However, after FCCP treatment, WT parkin appeared to be specifically recruited to mitochondria (Fig. 5A), as previously reported^26,27^. Redistribution of WT parkin from the cytosol to the mitochondria after FCCP treatment was confirmed by mitochondria isolation experiments (Fig. 5B). Presence of parkin in cytosol or mitochondria fractions was analyzed by immunoblotting with anti-EGFP antibody and normalized to caspase 3 or TOM20, respectively. The enrichment of parkin was determined by calculating the ratio between the amount of recovered parkin in mitochondria fraction and the amount of recovered parkin in the cytosol fraction. The amount of WT parkin in the mitochondria fraction significantly increased when the mitochondria membrane was depolarized (Fig. 5B; p = 0.037). Results concerning N52Mfs*29 indicate that this variant does not completely abolish the redistribution of parkin to depolarized mitochondria. It was possible to observe some overlap of parkin with mitochondria with and without FCCP treatment (Fig. 5A), and there was an increase of parkin in the mitochondria fraction after FCCP treatment (Fig. 5B; however, this increase was not significant (p = 0.056). In contrast, the L358Rfs*77 variant impaired mitochondria redistribution of parkin (Fig. 5A,B). Distribution of parkin did not change after FCCP treatment (Fig. 5A), neither did the amount of parkin in the mitochondria fraction (Fig. 5B).

## Discussion

The autosomal recessive inheritance of parkin-related PD suggests that protein loss-of-function is at the basis of ARJP pathogenesis. Functional studies reported that even variants that do not affect parkin E3 ligase activity, manifest loss-of-function by causing defects in protein folding, solubility, aggregation, localization or targeting of proteins for proteasome degradation^19–21^. Furthermore, despite the high number of parkin variants identified to date (about 170), the pathogenic relevance remains unclear for several of the variants^3,20^.

In this study, we determined the pathogenic mechanisms underlying two frameshift parkin variants. The N52Mfs*29 variant was already reported and is highly prevalent in the Portuguese and Spanish population^7,28^, while the L358Rfs*77 variant was recently identified in the Portuguese population^7^. Both variants seemed to reduce protein stability, since both were prematurely degraded by the proteasome (Fig. 1C). The low expression of these variants supports the theory of a loss-of-function phenotype^19,29^. However, both protein variants were still expressed at moderate levels, detected by immunoblotting and immunofluorescence, when overexpressed in cultured cells. This raised the question whether N52Mfs*29 and L358Rfs*77 endogenous proteins could be fully degraded by the proteasome, what would be interesting to study in patients’ cells. Nevertheless, since both overexpressed variants were present in protein aggregates (Fig. 3A), the ability of the proteasome system to fully degrade them could be compromised.

Additionally, it is also important to understand if N52Mfs*29 and L358Rfs*77 variants impair the molecular properties and enzymatic activity of parkin. The domain structure of parkin regulates its enzymatic activity. Resolution of the crystal structure showed that parkin exists in an autoinhibitory state. The E2 binding site on the RING1 domain is occluded by the UBL domain and the REP linker, while the catalytic site in the RING2 domain is blocked by the RING0 domain^8–10^. Therefore, activation of parkin requires additional conformational changes to bring the RING2 and E2 active sites closer for ubiquitin transfer. The activation process requires binding of phosphorylated ubiquitin generated by PINK1, which decreases the association of the UBL domain with the RING1 domain. Thus, PINK1 is able to efficiently phosphorylate the UBL domain of parkin, which then binds to the RING0 domain and releases the catalytic RING2 domain^30^.

The N52Mfs*29 and L358Rfs*77 variants lack important parkin domains, which can cause not only disruption of parkin folding, but also misregulation of parkin autoinhibition and enzymatic functions^20^. Moreover, the sequence of N52Mfs*29 lacking the Ser65 residue that is phosphorylated by PINK1^12^, implies that this variant confers distinct molecular properties to parkin. Indeed, we found that N52Mfs*29 caused protein misfolding leading to the formation of protein aggregates and altered subcellular distribution (Fig. 3). Thereby, decreased solubility of this variant (Fig. 2) may be explained not only by the presence of parkin in detergent-insoluble aggregates, but also by its higher degree of association with membranes (Fig. 3). Given the protein sequence of this variant expressing only the UBL domain and resembling ubiquitin, this phenotype is not surprising. The wide distribution throughout the cell and accumulation on aggregates is typical of ubiquitin. Furthermore, N52Mfs*29 showed reduced self-ubiquitination activity (Fig. 4A), which could result from either high turnover of the protein or impaired enzymatic activity^21^. Analysis of parkin ability to ubiquitinate a substrate in the presence of a proteasome inhibitor showed that the N52Mfs*29 variant impaired parkin enzymatic activity, as it was not able to significantly ubiquitinate p62 substrate (Fig. 4B). Nonetheless, this variant was still able to relocalize to depolarized mitochondria (Fig. 5A), although this was not significant (Fig. 5B). Once again, it seems that N52Mfs*29 mimics ubiquitin properties, which also concentrates on depolarized mitochondria^15^. Therefore, we believe that N52Mfs*29 variant also impairs parkin/PINK1-mediated mitophagy, as described for other parkin variants^25,26^.

Interestingly, the L358Rfs*77 variant also affected protein folding since it caused protein aggregation and, thus, decreased solubility (Fig. 2), and redistribution of parkin mainly to the nucleus (Fig. 3). Other variants that lack the C-terminal domain of parkin have also been reported to cause misfolding and aggregation, resulting in altered subcellular distribution^19,21,31^. Indeed the C-terminal domain of parkin is indispensable for proper folding as shown on crystal structure of parkin. Moreover, this variant lacks the catalytic RING2 domain located in the C-terminal^8–10^. Nonetheless, redistribution of parkin to the nucleus has not been reported for other variants. We checked the additional 77 amino acids of L358Rfs*77 for the presence of a nuclear localization signal, but we could not find any using bioinformatics analysis. Thereby, it would be interesting to study if any particular loss/gain of protein interactions and/or post-translational modifications are responsible for targeting this protein to the nucleus. Nonetheless, L358Rfs*77 retained the capacity of self-ubiquitination, as shown by the presence of high molecular weight species of ubiquitinated parkin (Fig. 4A). This could indicate either maintenance of enzymatic activity or accumulation of the ubiquitinated protein that was not completely degraded by the proteasome. To account for the potential mechanisms by which L358Rfs*77, with apparent increased ubiquitination activity, showed a loss-of-function phenotype (Figs 2, 3), we studied the capacity of this protein to ubiquitinate a substrate and redistribute to depolarized mitochondria. Our results, confirmed that L358Rfs*77 was not able to significantly ubiquitinate p62, neither redistribute to depolarized mitochondria. Reduced ability to ubiquitinate substrates could also be the result of loss of binding to substrates, coenzymes or adaptor proteins. This does not seem to be the case for L358Rfs*77, since it was able to bind p62 substrate (Fig. 4B), thereby showing that this variant impaired parkin enzymatic activity and mediated-mitophagy. Other truncating variant (W453*) has also been reported to retain self-ubiquitination activity, but not the ability to ubiquitinate substrates^13,21,26^. Nevertheless, further studies are needed to fully address potential mitophagy defects caused by N52Mfs*29 and L358Rfs*77 variants. Introducing parkin variants in cellular models by gene-editing technologies would be of great potential to study mitophagy impairment.

Ultimately, despite variable molecular, binding and ubiquitination properties, all parkin variants eventually lead to impaired degradation of substrates, leading to an abnormal accumulation that may be toxic to cells. In addition, intriguing data suggests that some parkin variants may be toxic to cells (Wang, Lu *et al*. 2007).

Despite being highly prevalent in the Portuguese and Spanish populations (Munoz, Tolosa *et al*. 2002, Morais, Bastos-Ferreira *et al*. 2016), the biochemical characteristics of the N52Mfs*29 variant in PD pathology had not been clarified so far. The same happens for the L358Rfs*77 variant that was recently identified in the Portuguese population (Morais, Bastos-Ferreira *et al*. 2016). The results of our study imply a causative role for the N52Mfs*29 and L358Rfs*77 variants in PD pathogenesis. It was shown that these parkin truncating variants have different biochemical properties, are abnormally localized and lack enzymatic activity. Therefore, loss-of-function activity may be explained not only by loss of parkin domains and disruption of protein folding, but also by mislocalization of parkin from its regular cytoplasmic distribution and site of enzymatic activity. Accumulation of parkin substrates and damaged mitochondria have been observed in other models of parkin loss-of-function, which may ultimately lead to neurodegeneration.

## Methods

### Antibodies

Primary antibodies: mouse monoclonal anti-EGFP antibody (Abnova, MAB1765), mouse monoclonal anti-GFP antibody (Rockland, 600-301-215), mouse monoclonal anti-beta-actin (Sigma-Aldrich, A5441), mouse monoclonal anti-GM130 (BD Biosciences, 610822), mouse monoclonal anti-caspase 3 (Cell Signaling, 9668), mouse monoclonal anti-HDAC2 (Santa Cruz Biotechnology, sc-9959), rabbit polyclonal anti-histone H3 (Abcam, ab1791), mouse monoclonal anti-p62 (Proteintech, 66184-1-Ig), mouse monoclonal anti-TOM20 (BD Biosciences, 612278). Secondary antibodies: horseradish peroxidase (HRP)-conjugated goat anti-mouse IgG (Santa Cruz Biotechnology, sc-2005), HRP-conjugated goat anti-rabbit IgG (Calbiochem, Merck-Millipore, 401393), mouse TrueBlot^TM^ ULTRA (Rockland, 18-8817-33).

### Expression vectors

The pEGFP-C1-Parkin-WT plasmid was kindly provided by Dr. Kawajiri, S. (Juntendo University School of Medicine, Tokyo, Japan). This plasmid was modified by site-directed mutagenesis using the QuikChange II Kit (Agilent) to produce disease-associated parkin plasmids. The following primers pairs were used to introduce N52Mfs*29 and L358Rfs*77 variants: forward primer 5′-GGGAAGGAGCTGAGGATGACTGGACTGTGC-3′ and reverse primer 5′-GCACAGTCCAGTCATCCTCAGCTCCTTCCC; and forward primer 5′- CGAAGGGGGCAATGGCAGGGCTGTGG and reverse primer 5′-CCACAGCCCTGCCATTGCCCCCTTCG, respectively. pRK5-HA-ubiquitin was a gift from Ted Dawson (Addgene plasmid 17608)^17^.

### Cell culture and transfection

HEK293T cells (kindly provided by Dr. Elsa Logarinho, IBMC/i3S, Porto) were grown in DMEM high glucose GlutaMAX™ supplemented with 10% fetal bovine serum (FBS) and 1% antibiotic-antimycotic (Gibco, Life technologies) at 37 °C in a humidified 5% CO_2_ atmosphere. Cells were transiently transfected for 24 h with each plasmid using jetPRIME (Polyplus-transfection) according to the manufacturer’s protocol.

In order to inhibit/enhance different proteolytic pathways, cells were treated at 24 hours post transfection with the following drugs for 18 hours: the proteasome inhibitor MG132 (5 μM, Calbiochem, EMD Millipore), autophagy inducer rapamycin (200 mM, Calbiochem, EMD Millipore), ERAD inhibitor EerI (10 µM, Calbiochem, EDM Millipore) or control vehicle DMSO (Sigma-Aldrich), and the lysosomotropic agent NH4Cl (15 mM, Sigma-Aldrich) or control vehicle DMEM (Gibco, Life technologies).

To induce mitochondria membrane depolarization, cells were treated at 24 hours post transfection with the mitochondria uncoupler carbonyl cyanide p-(tri-fluromethoxy)phenyl-hydrazone (FCCP, 30 µM) or with the control vehicle DMSO for 1 hour.

### Western blot analysis

Cells expressing target proteins were collected in RIPA buffer (Sigma; 150 mM NaCl, 1.0% IGEPAL® CA-630, 0.5% sodium deoxycholate, 0.1% SDS, 50 mM Tris, pH 8.0) supplemented with cOmplete Protease Inhibitor Cocktail (Roche) and then sonicated. Total protein concentration was measured with the Pierce BCA protein assay kit (Thermo Scientific) according to manufacturer’s instructions. Samples (30–50 µg of total protein) were separated on SDS-PAGE and electrophoretically transferred onto PVDF membranes (Merck-Milipore) using a wet electroblotting system (Bio-Rad). Membranes were blocked in 5% non-fat dry milk in PBS-T for 1 hour, at room temperature (RT), and subsequently incubated with specific primary antibodies, as indicated (diluted in 3% non-fat dry milk in PBS-T), overnight at 4 °C, except for beta-actin antibody that was incubated for 1 hour at RT. The membranes were washed with PBS-T and then incubated with HRP-conjugated secondary antibody for 1 hour at RT. Following three washes with PBS-T, detection was achieved using WesternBright^TM^ Sirius or WesternBright^TM^ ECL- HRP substrates (Advansta) and chemiluminescence detected with a ChemiDoc^TM^ XRS+ Imaging System (Bio-Rad). Protein bands were quantified using the Image Lab^TM^ 5.2.1 Software (Bio-Rad). Quantitative comparisons between samples of each experiment were always performed on the same blot. When necessary, membranes were stripped by incubation in stripping solution (62.5 mM Tris-HCl pH 6.7, 2% SDS, 100 mM beta-mercaptoethanol) at 50 °C for 30 min, with gentle agitation.

### Detergent solubility assay

Cells expressing target proteins were washed with PBS and then collected in 0.1% Triton X-100 (Merck-Millipore) in 1x PBS supplemented with cOmplete Protease Inhibitor Cocktail (Roche). Homogenates were centrifuged at 15000 g for 20 min at 4 °C to separate the supernatant (soluble) and pellet (insoluble) fractions. Pellet fractions were solubilized in 1x laemmli buffer and supernatants were brought to the same volume and 1x concentration by adding 6x laemmli buffer, and both fractions were then boiled at 95 °C for 10 min. To compare the relative distribution of parkin, equal volumes pellet and supernatant were loaded on SDS-PAGE [adapted from^19^].

### Subcellular protein fractionation and mitochondria isolation

For subcellular protein fractionation, cells expressing target proteins were washed and scrapped from the plate in ice cold 1x PBS. The whole cell extract (corresponding to total fraction) was centrifuged at 500 g for 3 min and the supernatant discarded. Subcellular protein extraction was then performed, using the Subcellular Protein Fractionation Kit for cultured cells (Thermo Scientific), according to the manufacturer’s instructions. Subcellular fractions corresponding to cytoplasm, membranes, soluble nuclear and chromatin-bound nuclear extracts were isolated.

For mitochondria isolation, cells expressing target proteins were washed and scrapped from the plate in ice cold 1x PBS. The whole cell extract (corresponding to total fraction) was centrifuged at 850 g for 2 min and the supernatant discarded. Mitochondria isolation was then performed using the Mitochondria Isolation Kit for cultured cells (Thermo Scientific), according to the manufacturer’s instructions. For obtaining the mitochondria pellet, centrifugation was performed at 12000 g for 15 min at 4 °C. The mitochondria pellet was lysed in 1% triton X-100 in TBS (25 mM Tris, 0.15 M NaCl, pH 7.2), supplemented with cOmplete Protease Inhibitor Cocktail (Roche). Subcellular fractions corresponding to cytosol and mitochondria-enriched fractions were isolated.

### Co-immunoprecipitation and ubiquitination assays

Cells were collected in lysis buffer (50 mM Tris-HCl pH 8.0, 120 mM NaCl, 0.5% Triton X-100) supplemented with the Pierce Protease and Phosphatase Inhibitor tablets (Thermo Scientific) and sonicated. Dynabeads^TM^ M-280 Sheep Anti-Mouse IgG (Life Technologies) were washed in 3% BSA/PBS. GFP or p62 antibodies were coupled to Dynabeads (1 µg antibody/50 µl beads), by incubating with rotation overnight at 4 °C. Cell lysates were precleared with 5 μL Dynabeads for 1 hour at 4 °C and then incubated with antibody-Dynabeads with rotation overnight at 4 °C. The immunoprecipitates were washed in 3% BSA/PBS and then in PBS, and further transferred to a clean tube. The proteins were eluted by boiling in 1x laemmli buffer at 90 °C for 10 min.

### Immunofluorescence

Cells expressing target proteins were fixed using 4% paraformaldehyde/4% sucrose for 20 min and permeabilized with 0.3% triton X-100 for 20 min. After washing with PBS, cells were blocked in 3% BSA/PBS for 45 min and further incubated with the primary antibodies overnight at 4 °C. Then, cells were incubated with the secondary antibodies for 1 hour at RT, washed with PBS and stained with Hoechst 33342 (Life Technologies) for 5 min to label the nucleus. Preparations were mounted with ProLong^TM^ Gold Antifade Mountant (Life Technologies) and visualized using an epifluorescence Zeiss Axio Imager Z1 microscope equipped with an Axiocam MR3.0 camera and Axiovision 4.7 software.

### Statistical analysis

Analyses of data were performed using the IBM SPSS Statistics 25.0 software. All quantitative data are expressed as mean ± standard deviation (SD) of at least three independent experiments. Statistical significance analysis was conducted using one-way ANOVA with Tukey post-hoc test, when comparing means of more than two groups, or two-tailed Student’s t-test to compare means between two groups, the level of statistical significance being set at p < 0.05.

## Supplementary information

Additional information
