# Supplementary material for: Parkin truncating variants result in a loss-of-function phenotype
Source: Sci Rep. 2019 Nov 6;9:16150. doi: 10.1038/s41598-019-52534-6 (PMC6834586; doi:10.1038/s41598-019-52534-6)

**Parkin truncating variants result in a loss-of-function phenotype**

Mariana Santos^1*^, Sara Morais^1^, Conceição Pereira^1^, Jorge Sequeiros^1,2,3^, Isabel Alonso^1,2^

^1^UnIGENe, IBMC - Institute for Molecular and Cell Biology, i3S - Instituto de Investigação e Inovação em Saúde, Univ. Porto, Portugal

^2^CGPP, IBMC - Institute for Molecular and Cell Biology, i3S - Instituto de Investigação e Inovação em Saúde, Univ. Porto

^3^ICBAS - Instituto de Ciências Biomédicas Abel Salazar, Univ. Porto, Portugal

*mariana.graca@ibmc.up.pt


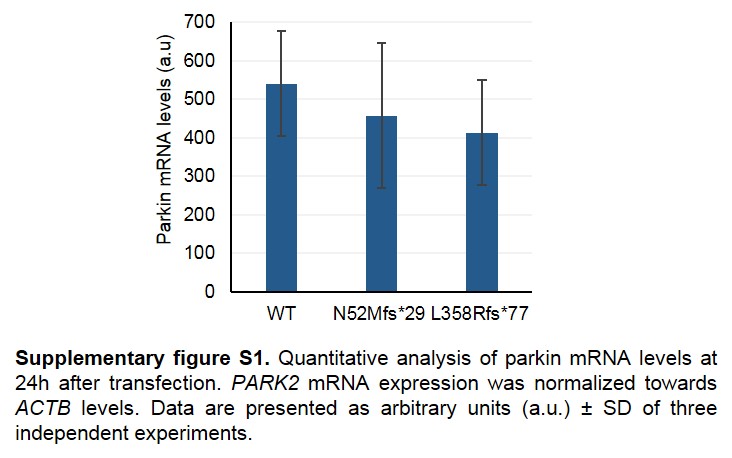


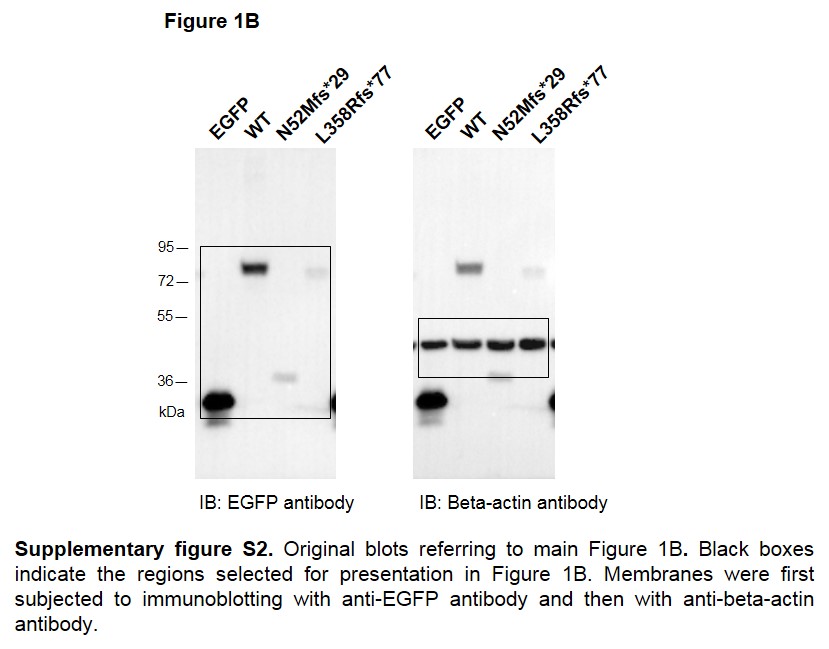


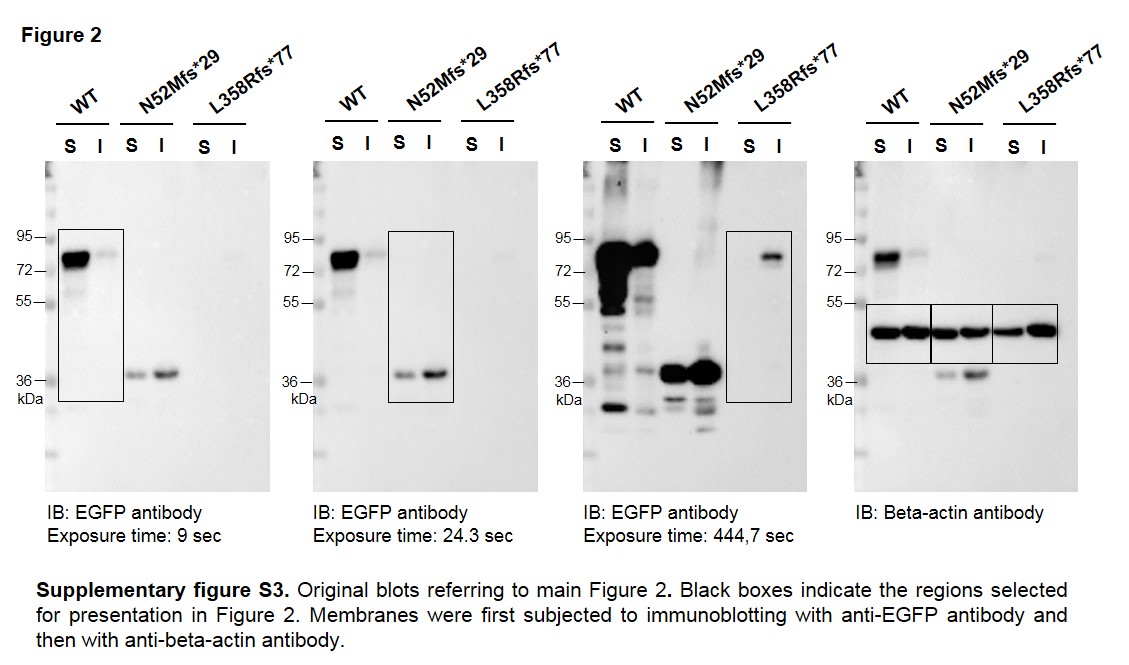


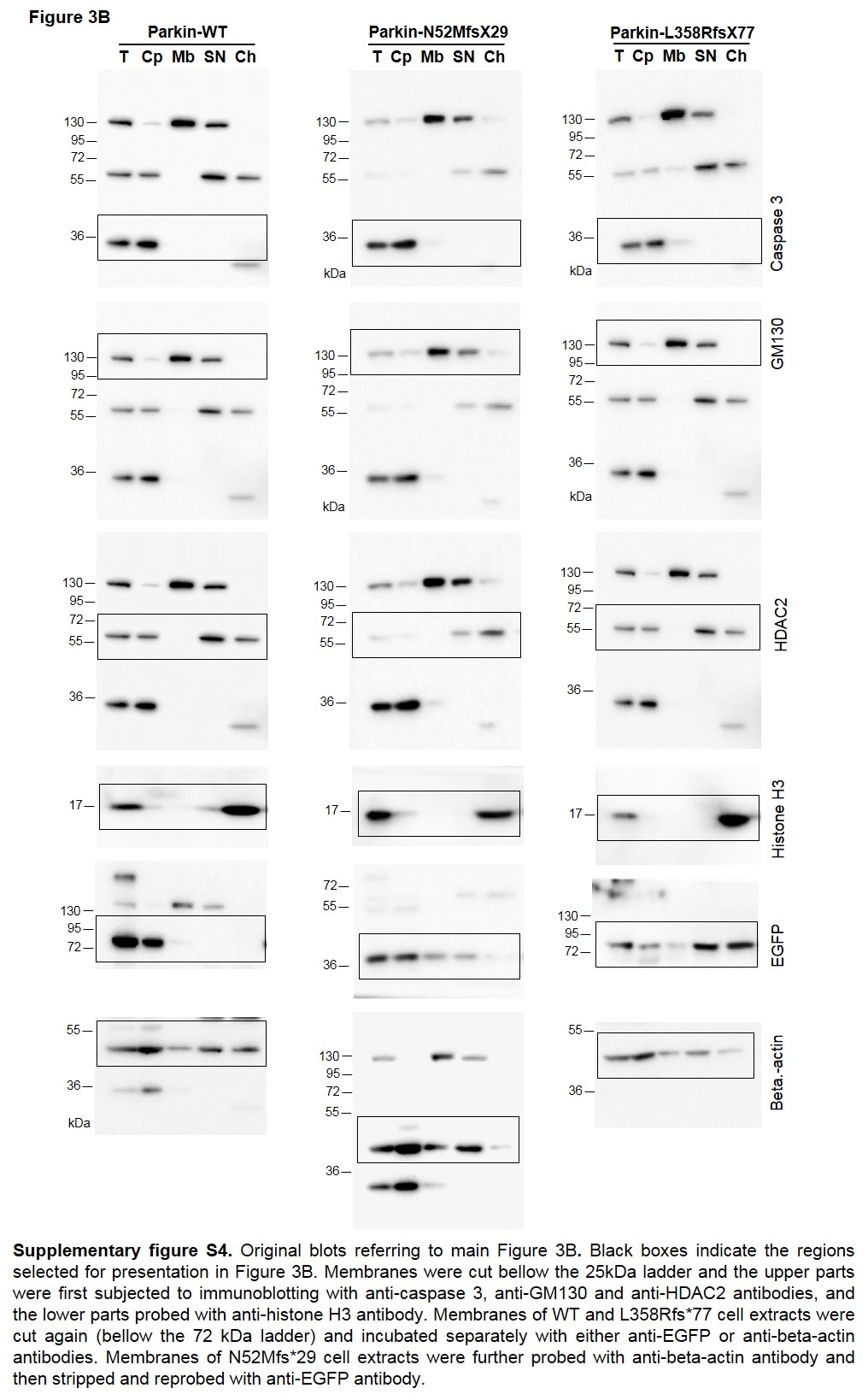


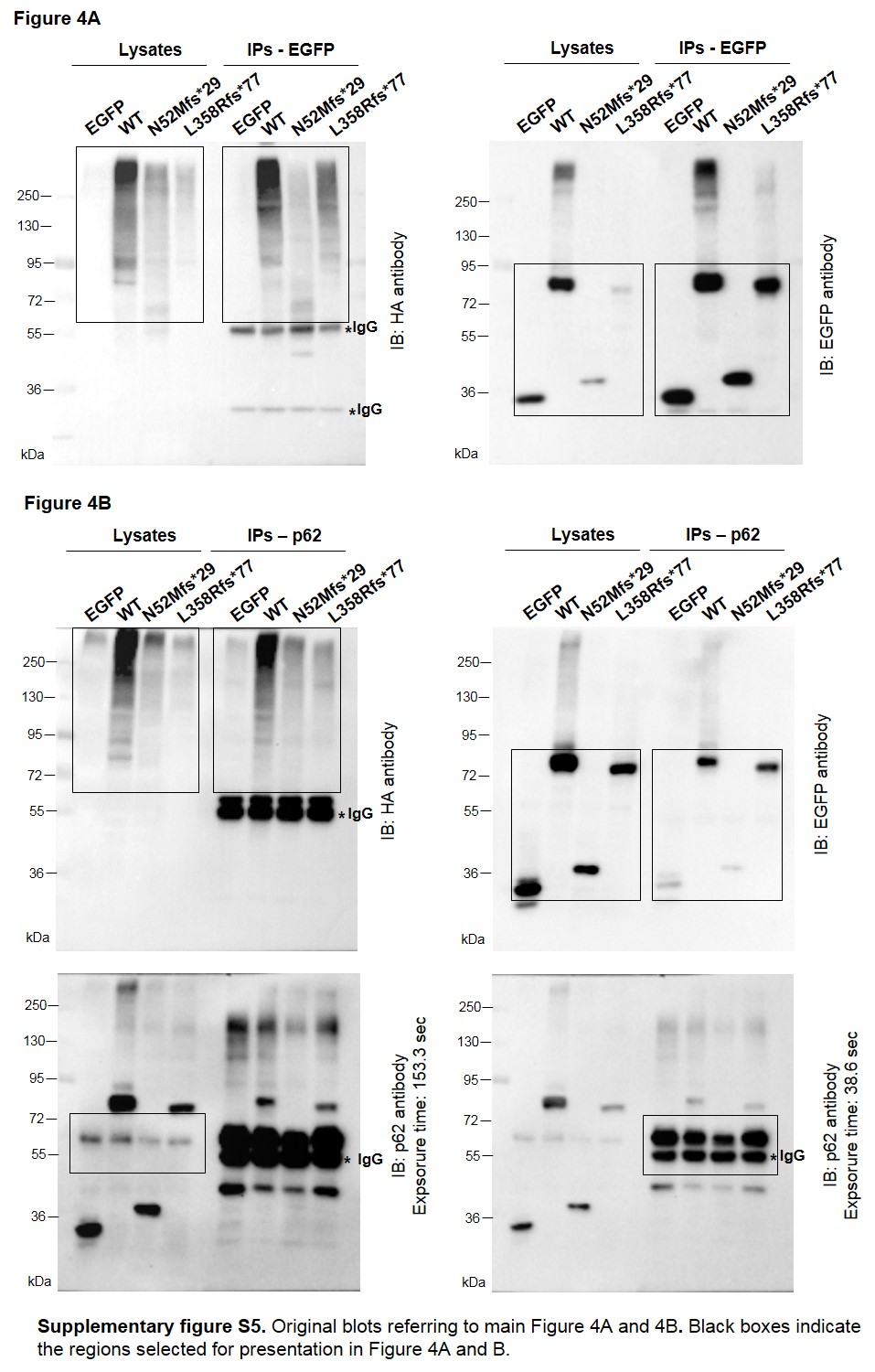


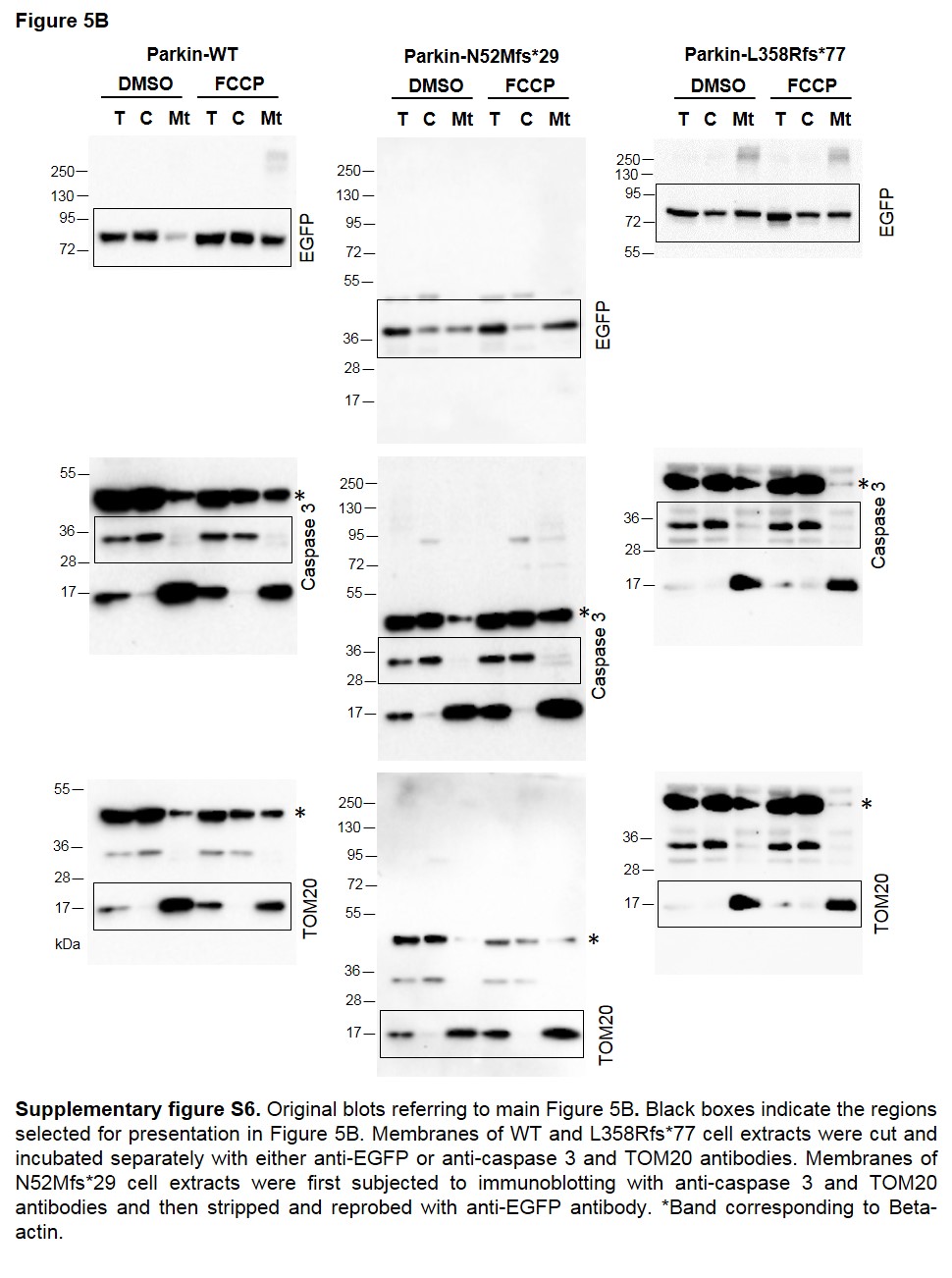

Supplement: Supplementary file 1 — Additional information [file 41598_2019_52534_MOESM1_ESM.docx]
